# The effects of centipedegrass extract on hair growth via promotion of anagen inductive activity

**DOI:** 10.1371/journal.pone.0265532

**Published:** 2022-03-23

**Authors:** Fatuma Jumapili Ramadhani, Dong-Ho Bak, Seong Hee Kang, Chul-hong Park, Sang hwa Park, Byung Yeoup Chung, Hyoung-woo Bai

**Affiliations:** 1 Research division for Biotechnology, Advanced Radiation Technology Institute (ARTI), Korea Atomic Energy Research Institute (KAERI), Jeongeup, Korea; 2 Radiation Biotechnology and Applied Radioisotope Science, University of Science and Technology (UST), Daejeon, Korea; 3 Bacteria Research Team, Nakdonggang National Institute of Biological Resources (NNIBR), Sangju, Korea; Chonnam National University Medical School, REPUBLIC OF KOREA

## Abstract

To investigate the CGE on hair growth and to explore the mechanism that is involved in the acceleration of anagen induction, we investigated the effects of CGE studied on cell proliferation and molecular mechanism in human hair dermal papilla cells (hDPCs) and keratinocytes (HaCaT cells). Additionally, hair growth evaluation was carried out following topical treatment of the dorsal skin of telogen C57BL/6 mice with CGE for 14 days. As result, CGE increased cell viability and ALP activity in hDPCs. Moreover, CGE increased the expression of catenin beta 1 (CTNNB1), ALP, sex-determining region Y-box 2 (SOX2), insulin-like growth factor 1 (IGF1), and vascular endothelial growth factor A (VEGFA) genes in hDPCs. CGE increased the expression of proteins such as ALP, β-catenin, and phosphorylation of glycogen synthase kinase 3β (pGSK3β), and protein kinase B (pAKT) in hDPCs. Furthermore, CGE induced the proliferation of HaCaT cells and up-regulated AKT-ERK-GSKβ-β-catenin signaling in HaCaT cells. Additionally, the anagen induction effects of CGE were confirmed on the telogen-anagen transition mice model. these findings demonstrated that CGE promoted the entering the growth phase of hair follicle via activation of β-catenin signaling pathways in vivo. Thus, this study suggests that CGE might be a potential therapeutic reagent for hair growth.

## Introduction

Centipedgrass is a medicinal plant found in Southeast Asia, rich in C-glycoside flavones, mainly maycin and its derivatives. Centipedegrass extract (CGE) is known to have different biological properties linked to anti-insecticide, anti-bacterial, and anti-fungal activities [1]. Furthermore, Badaboina et al. reported that CGE accelerated the apoptosis of skin melanoma cells by stopping cell division at the G2/M phase [2]. On the other hand, CGE has a free radical scavenger activity, which had been suggested a role as a skin protective agent [3]. However, the functions of CGE on anagen phase restoration and hair regrowth have not been elucidated yet.

The hair follicle (HF) has preserved unique characteristics by undergoing cyclic restoration in three specific stages known as anagen, catagen, and telogen [4]. Interfering with the transition of the telogen to anagen phase delays the hair cycle, inhibits hair shaft growth, and thins the hair, blocking new hair restoration [5]. Hair loss has been shown to impact the psychological well-being of individuals by causing anxiety and depression [6]. Pharmaceutical therapeutics, such as vasodilators (minoxidil) and 5α-reductase inhibitors (finasteride) have been approved for alopecia management. Nonetheless, the application of medical treatments may be limited due to some reversible effects. Minoxidil has been documented to cause dermal rashes, dryness, and weight gain [7,8]. Besides, finasteride affects libido in males and breast health in females [9]. Thus, an alternative and safe hair restoration therapy is highly demanded.

Hair regrowth and the periodic cyclic renewal of HF depend on Wnt/β-catenin signaling activation [10]. This pathway is activated when ligands and receptors interact to recruit the degradation complex (APC-GSK-3β-CK1-Axin), which helps stabilize β-catenin in the cytoplasm. Subsequently, the dimerization between β-catenin and LEF1/TCF occurs in the nucleus. On the other hand, reversing action occurs when DDK1 binds Wnt receptors and activates the degradation mechanism, which breaks down β-catenin in the cytoplasm. The inability of β-catenin to enter the nucleus inhibits the transcription of Wnt reactive genes [11]. After development, the Wnt/β-catenin pathway plays an essential role in the initiation of anagen and controls the life-long restoration of the HF cycle [12,13]. Furthermore, the Wnt/β-catenin pathway stimulates hair inductivity in hDPCs and increases signaling relevant to the anagen phase [14]. Therefore, the method through the regulation of β-catenin expression will be one of the effective hair loss treatment strategies. In this study, we aimed to examine whether the CGE could efficiently promote anagen inductive properties in hDPCs and HaCaT cells. Also, we analyzed the telogen to anagen transition to confirm by which the effects of CGE on hair re-growth in vivo.

## Material & methods

### Preparation of the CGE

Centipedegrass seeds imported from the Fukukaen Nursery (Blu co. Ltd., Nagoya, Japan) were in 2010 cultivated at the Korea Atomic Energy Research Institute (KAERI, Jeongeup, South Korea). The leaves of centipedegrass were harvested in October 2011 and stored at −80°C until use. The extraction method was performed as previously reported [15]. The dried centipedegrass (5 kg) leaves were ground in a Wiley mill and passed through a 420-μm sieve. The ground sample (1 kg) was extracted 3 times with 80% methanol (MeOH, 100 l; Merck & Co, Inc., Whitehouse Station, NJ, USA) for 24 h with constant shaking at ambient temperature in the dark. The extracts were filtered using No. 2 filter paper (Advantec Mfs Inc., Dublin, CA, USA) and concentrated in vacuo. The MeOH extracts were fractionated with n-hexane and ethyl acetate (EA) (Merck & Co, Inc.). The EA extracts were concentrated in vacuo and the dried compounds were dissolved in MeOH. The dissolved extracts in MeOH were diluted in 20% MeOH and chromatographed over a Toyopearl HW-40C resin (Tosoh Co., Tokyo, Japan) column using 70% MeOH (elution volume, 700 ml). The fraction was evaporated and then freeze-dried. Dried extracts were reconstituted in dimethyl sulfoxide (DMSO) for cell treatment. After quantification of each of the five chemical components, including maysin and its derivative flavonoid compounds in the CG ethylacetate (EA-CG) fraction (e.g., luteolin, isoorientin, rhamnosylisoorientin, derhamnoslymaysin, and maysin), their production weight per 100 mg extracts was determined as the purification yield from each batch, as described in a previous report [16].

### Animals

All procedures for animal studies were reviewed and approved by the ARTI animal committee (IACUC NUMBER: KAERI-IACUC-2018-002). The design and all experimental procedures complied with animal laboratory sections. The 6-week-old female C57BL/6 mice were purchased from Orient Bio (Seoul, South Korea). The treatments were divided into 3 groups; 1) the control group (50% glycerol, 25% ethanol, and distilled water), 2) the positive control group was applied with 5% minoxidil (Hyundai Pharmaceutical Co Ltd, Cheonan, South Korea), and 3) CGE (1% CGE; 1% in 50% glycerol, 25% ethanol, and distilled water) group. Mice received the topical application of each treatment for 14 days. On the 14th day after hair removal, the dorsal skin was harvested for histological analysis at the end of the observation period.

### Histological analysis and immunostaining

The staining method was performed with some modifications in the previous report [17]. For Immunohistochemistry (IHC), we used antibodies against anti-β-catenin (1:1000, cat no. ab6302). Primary antibodies were detected via biotinylated secondary antibodies plus a streptavidin-peroxidase complex (Vector Laboratories, Inc., Burlingame, CA, USA) and brown FAST DAB (Thermo Fisher Scientific, Inc., Waltham, MA, USA) staining. Slides were observed via light microscopy (DM750, Leica, Wentzler, Germany). Antibodies against SHH (1:200, cat no.LS-C343862), alkaline phosphatase (ALP) (1:200, cat no. ab67228) and Ki-67 (1:1000, cat no.9027) were used for immunofluorescence (IF). Slides were mounted, and images were acquired using a confocal microscope (LSM700, Zeiss, Oberkochen, Germany).

### Cell proliferation assay

hDPCs were obtained from CEFO Co., Ltd. (cat. no. CB-HDP-001) and cultured for 6 passages before use in experiments. The experiments were conducted using short incubation periods as long in vitro culture resulted in hDPCs losing their original characteristics and functions. HaCaT cells, which are immortalized human keratinocytes, were obtained from Addexbio Technologies (cat. no. T0020001). Two cells were seeded in Dulbecco’s modified Eagle’s medium (DMEM; Invitrogen/Gibco-BRL), supplemented with 5% fetal bovine serum (FBS; Invitrogen/Gibco-BRL) and 1% penicillin, and cultured in a humidified environment. hDPCs and HaCaT cells were plated at a density of 1×10^4^/well and 0.5×10^4^/well, respectively, into 96-well plates and were continuously cultured for 24 h under each test condition. hDPCs and HaCaT cells were treated with vehicle control (Veh), 10 μM minoxidil, and different concentrations of CGE (3.1∼50 μg/mL) for 24 h. Next, 5 mg/mL MTT (20 μl) solution was added to each well at 37°C for 2 h, followed by 200 μl of DMSO to dissolve the insoluble formazan. The number of viable cells was quantified at 560 nm using the Tecan Elisa reader (Tecan Group Ltd. Seestrasse, Männedorf, Switzerland).

### Alkaline phosphatase activity

hDPCs (1.5×10^5^) were seeded into 6-well plates for 24 h, followed by treatment with Vehicle control (1% DMSO), minoxidil (10 μM), and CGE (3.1–50 μg/mL) for 24 h. ALP activity was analyzed using the SensoLyte^®^ pNPP Alkaline Phosphatase Assay Kit (Cat no. AS-72146, AnaSpec, Inc., Fremont, CA, USA) as previously described [18].

### Immunocytochemistry staining for Ki-67

Immunofluorescence assay was performed as previously described [19]. The cells were fixed in 4% formaldehyde and incubated in primary antibody anti-Ki-67 (1:1000, cat no.9027), the secondary antibody anti-rabbit IgG Alexa Fluor 555 conjugate (1:1000. Cat no. 4413, Cell signaling Technology, Beverly, MA, USA). 4′,6-diamidino-2-phenylindole (DAPI) staining was done by incubating the cells with Hoechst (1 μg/mL), and subjected to confocal microscopy by a confocal laser microscope (LSM 750; Zeiss, Heidelberg, Germany).

### Reverse transcription-quantitative polymerase chain reaction (RT-qPCR)

The total RNA was quantified by NanoDrop spectrophotometer (NanoDrop Technologies LLC, Wessington, South Dakota, USA). The laboPass^™^conversion kit (CosmoGENETECH, Seoul, South Korea) was used to synthesize the complementary DNA (cDNA). The resulting cDNA was subjected to real-time PCR using qPCR 2x PreMIX SYBR (Enzynomics, Seoul, Korea) and a CFX-96 thermocycler (Bio-Rad, Hercules, CA, USA). PCR conditions used to amplify genes were as follows: 10 min at 95°C and 40 cycles of 95°C for 10s, 60°C for 15s, and 72°C for 20s. Expression data were calculated from the cycle threshold value using the ΔCt method for quantification. Oligonucleotides used for real-time PCR were described in S1 Table.

### Western blot analysis

Western blot analysis was performed as previously reported [15]. primary antibodies at 1:1000 for anti-ERK, -phosphorylated ERK (pERK), -Akt, -phosphorylated Akt (pAkt),—GSK3β, -phosphorylated GSK3β (pGSK3β), -GAPDH were purchased from Cell signaling Technology (Beverly, MA, USA), and anti-β-catenin was obtained from Abcam (Cambridge, MA, USA) overnight at 4°C. The ECL reagents (Amersham Corp., Arlington Heights, IL, USA) were used to detect the protein bands, and images were captured using the iBright^™^ CL1000 imaging system (Thermo Fisher Scientific, Carlsbad, CA, USA).

### Statistical analysis

All data are presented as the mean ± standard deviation of three independent experiments. One-way analysis of variance (ANOVA) followed by Tukey’s test for post-hoc analysis was used to compare the differences between means. The value of P < 0.05 was considered to be statistically significant.

## Results

### Effects of CGE increased anagen inductive properties in hDPCs

**Fig 1:**
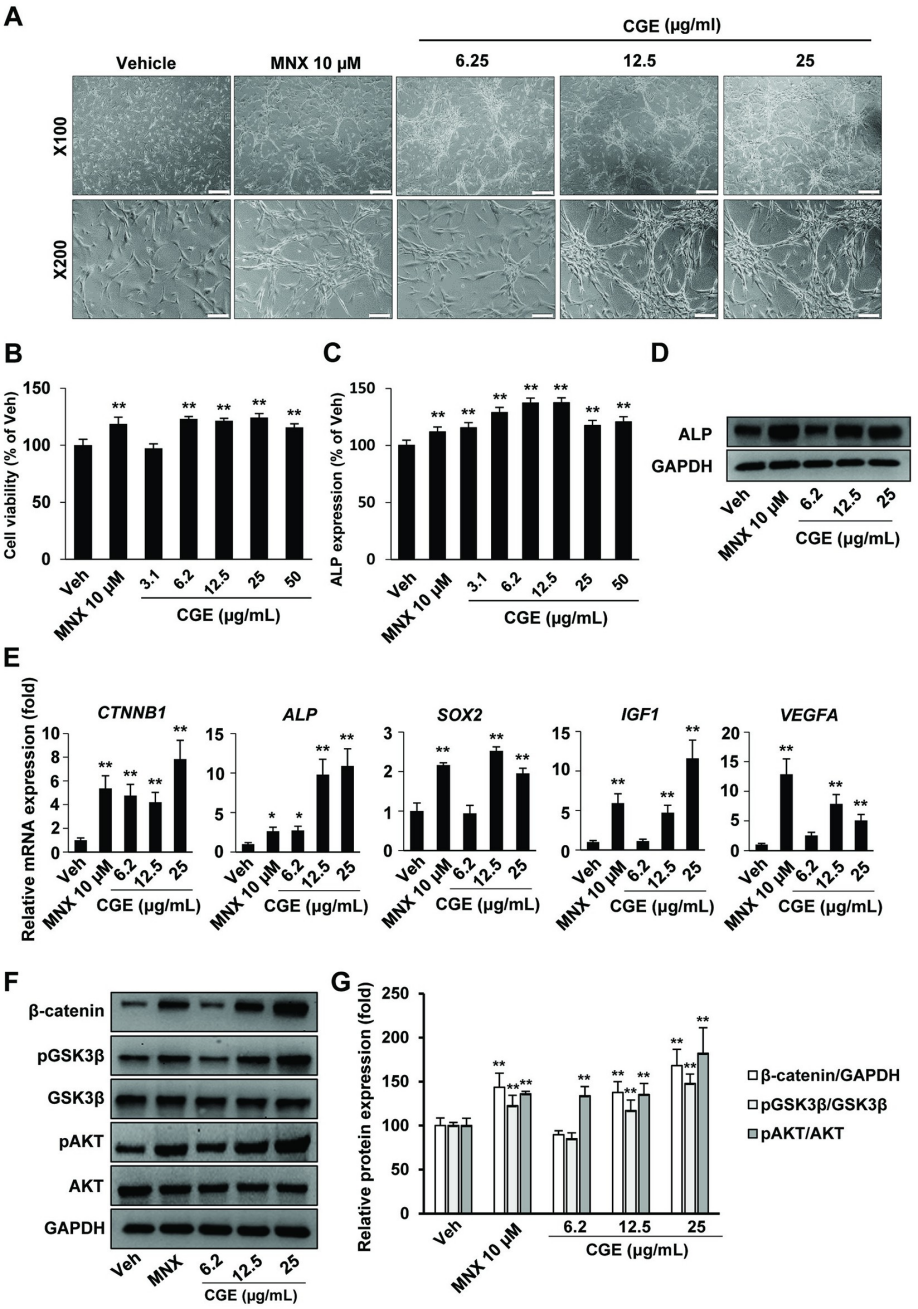
CGE increased anagen properties in hDPCs via up-regulating β-catenin signaling. (A) Morphological changes in hDPCs after CGE treatment. MNX used as a positive control. Scale bar: 200 μm (B) Cell viability. CGE treatment increased hDPCs viability at 24 h after treatment. CGE treatment increased ALP expression in hDPCs. ALP expression was measured by (C) ALP activity assay KIT and (D) western blot using anti-ALP. Anti-GAPDH used as a loading control. (E) mRNA transcription of CTNNB1, ALP, SOX2, IGF-1 and VEGF were measured by RT-qPCR after 12 h with each treatment. (F) Representative bands of Western blot analysis for the protein expression of β-catenin, pGSK3β, GSK3β, pAKT, AKT, or GAPDH antibodies. (G) Densitometry analysis of western blot. Data are presented as the mean ± SD. *p ≤ 0.05, **p ≤ 0.01 vs. Veh. Veh, vehicle control; MNX, minoxidil 10 μM; SD, standard deviation.

The aggregated morphology of hDPCs is considered to be associated with their hair inductive properties [20]. To assess the morphological changes in hDPCs, we treated with indicated concentration of Veh, MNX, and CGE for 24 h then observed with a microscope. Images were captured to observe the morphology of hDPCs. CGE treatments in hDPCs showed aggregation-like morphology in contrast to that in Veh-treated cells. The highest aggregation tendency was observed in 25 μg/mL CGE-treated cells (Fig 1A). To investigate whether CGE affected a proliferation of hDPCs directly, hDPCs were treated with different concentrations of CGE for 24 h, and cell viability was determined. Cell viability increased in 6.2 μg/mL, 12.5 μg/mL, 25 μg/mL or 50 μg/mL CGE treatment groups compared to vehicle control (Fig 1B). High expression of ALP is known to be one of the hair inductive properties during anagen phase [21]. In this study, results showed that CGE increased ALP expression compared to vehicle control (Fig 1C). In addition, western blot result also revealed an increase in the ALP expression levels by CGE treatments compared to Veh control (Fig 1D).

To further understand the mechanism of hair inductive activity in response to CGE treatment, we analyzed the genes-related to hair cycle progression in hDPCs after treatment with CGE for 24 h. As a result, CGE increased the transcription of *CTNNB1* and *ALP*. Importantly, CGE treatments increased transcription of hair growth-related factors—sex-determining region Y-box 2 (*SOX2*, a direct transcriptional regulator regulating the rate of HF growth in hDPCs), insulin-like growth factor 1 (*IGF-1*, *IGF-1* was shown to affect follicular proliferation, tissue remodeling, and the hair growth cycle, as well as follicular differentiation) and vascular endothelial growth factor (*VEGF*, *VEGF* is one of the most potent regulators of physiological and pathological angiogenesis and is an essential factor for hair growth) in hDPCs (Fig 1E)[13,22,23]. Additionally, CGE treatments increased β-catenin protein expression in a concentration-dependent manner. The level of pAKT increased in the treated groups compared to vehicle control. Similarly, pGSK3β increased in a concentration-dependent manner in CGE-treated groups (Fig 1F and 1G). These results suggested that CGE might promote the hair induction properties, necessary for hair cycle progression in hDPCs.

### CGE increased HaCaT cells proliferation via β-catenin signalling

**Fig 2:**
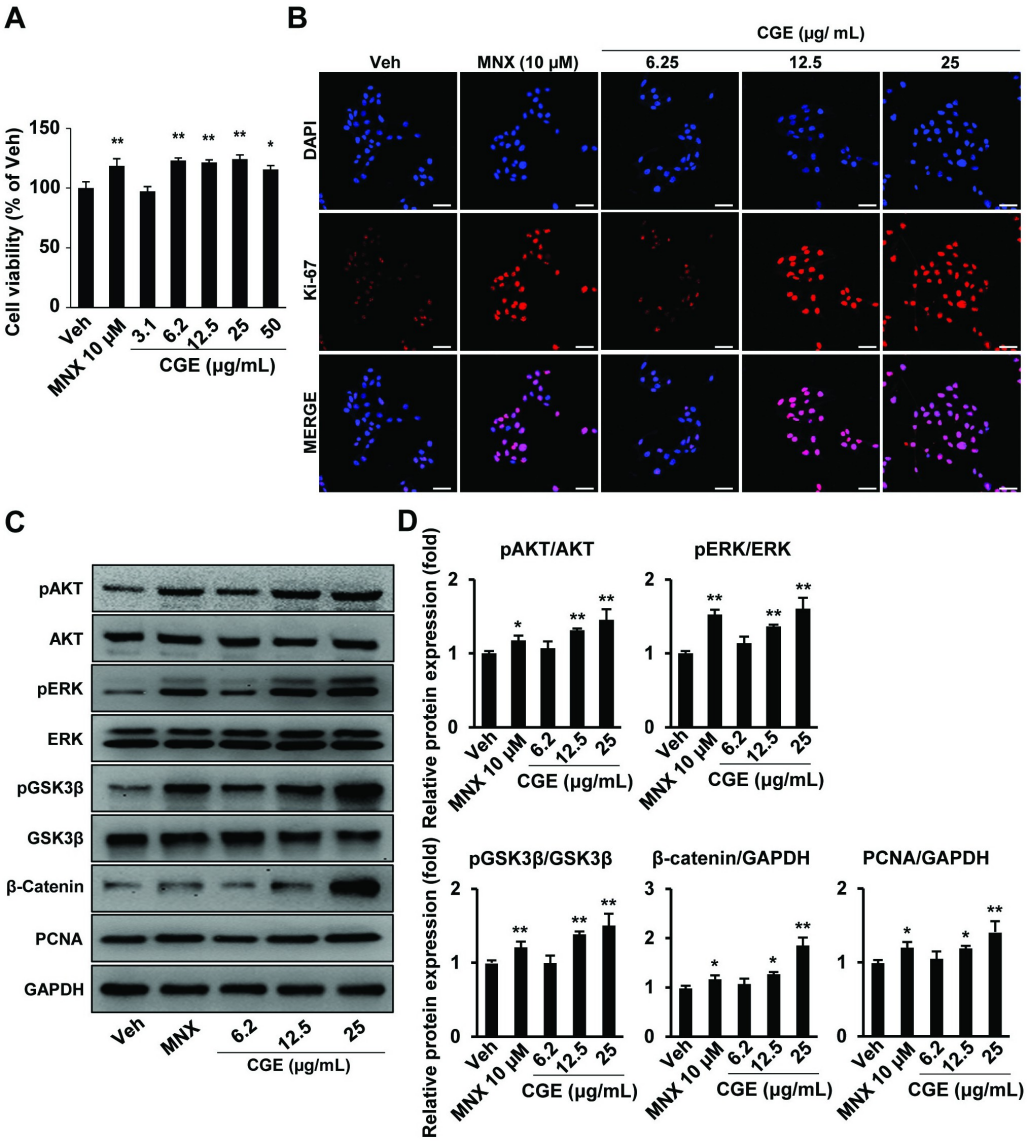
CGE increased HaCaT proliferation via up-regulating β-catenin signaling. (A) Cell viability. CGE treatment increased HaCaT viability after 24 h. (B) Representative images of Ki-67 expression. Scale bar: 50 μm (C) Representative images of Western blot analysis for the protein expression of β-catenin, pGSK3β, GSK3β, pAKT, AKT, pERK, ERK, PCNA, or GAPDH antibodies. (D) Densitometry analysis of western blot. Data are presented as the mean ± SD. *p ≤ 0.05, **p ≤ 0.01 vs. Veh. Veh, vehicle control; MNX, minoxidil 10 μM; SD, standard deviation.

To determine the role of CGE treatment on HaCaT cell proliferation, we conducted MTT assay on HaCaT cells treated with various concentrations of CGE for 24 h. The CGE-treated groups showed a significant increase in the viability of HaCaT cells compared to Veh group (Fig 2A). For further investigation, we decided to use 6.25 μg/mL, 12.5 μg/mL, 25 μg/mL concentrations of CGE for the following experiment. ICC was conducted for observing Ki-67 to determine the effects of CGE on keratinocyte proliferation. HaCaT cells received the indicated concentration of the Veh, MNX, and CGE for 24 h. Results showed that CGE- and MNX-treated groups were enhanced nuclear localization of Ki-67 compared to the Veh group. A higher number of Ki-67 positive cells was found at 12.5–25 μg/mL of CGE compared to the Veh group (Fig 2B). To further understand the mechanism of HaCaT proliferation in response to CGE, we observed the proteins related to hair cycle progression in HaCaT cells after treatment with MNX and CGE for 24 h. A previous report revealed that ERK1/2 and AKT pathways modulate keratinocyte proliferation [24]. In this study, results showed that β-catenin expression was increased in a concentration-dependent manner in CGE treated groups. Furthermore, the level of pAKT and pERK expression was increased in CGE-treated groups compared to the Veh group. Moreover, pGSK3β and proliferating cell nuclear antigen (PCNA, a cellular marker for proliferation) expression were increased in CGE treatment groups in a concentration-dependent manner (Fig 2C and 2D). These results suggested that CGE promoted keratinocyte proliferation, the prerequisite for hair cycle progression.

### CGE accelerated the telogen to anagen transition in C57BL/6 mice

**Fig 3:**
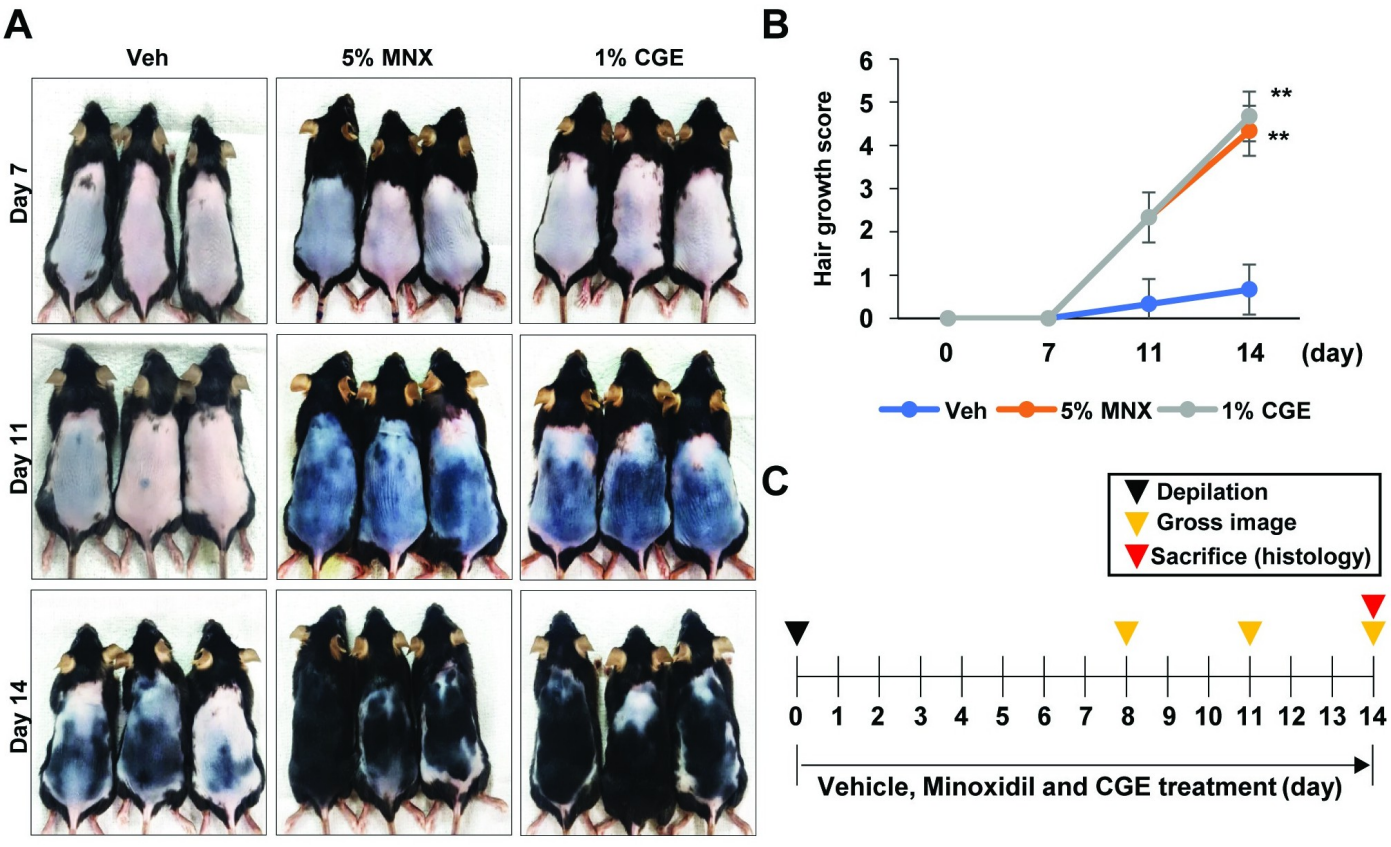
CGE promoted hair re-growth in C57BL/6 dorsal skin. Each candidate was applied to the back skin for 14 days. 5% minoxidil was used as a positive control. After depilation, the observation was preceded for 14 day, at the end of experiment, the sacrifice was conducted. (A) Gross images, at 7^th^, 11^th^, and 14^th^ day after CGE treatment. (B) Gross images were measured for hair growth score. (C) Schematic diagram of the experimental schedule. Data are presented as the mean ± SD. **p ≤ 0.01 vs. Veh. Veh, vehicle control; MNX, minoxidil 10 μM; SD, standard deviation.

**Fig 4:**
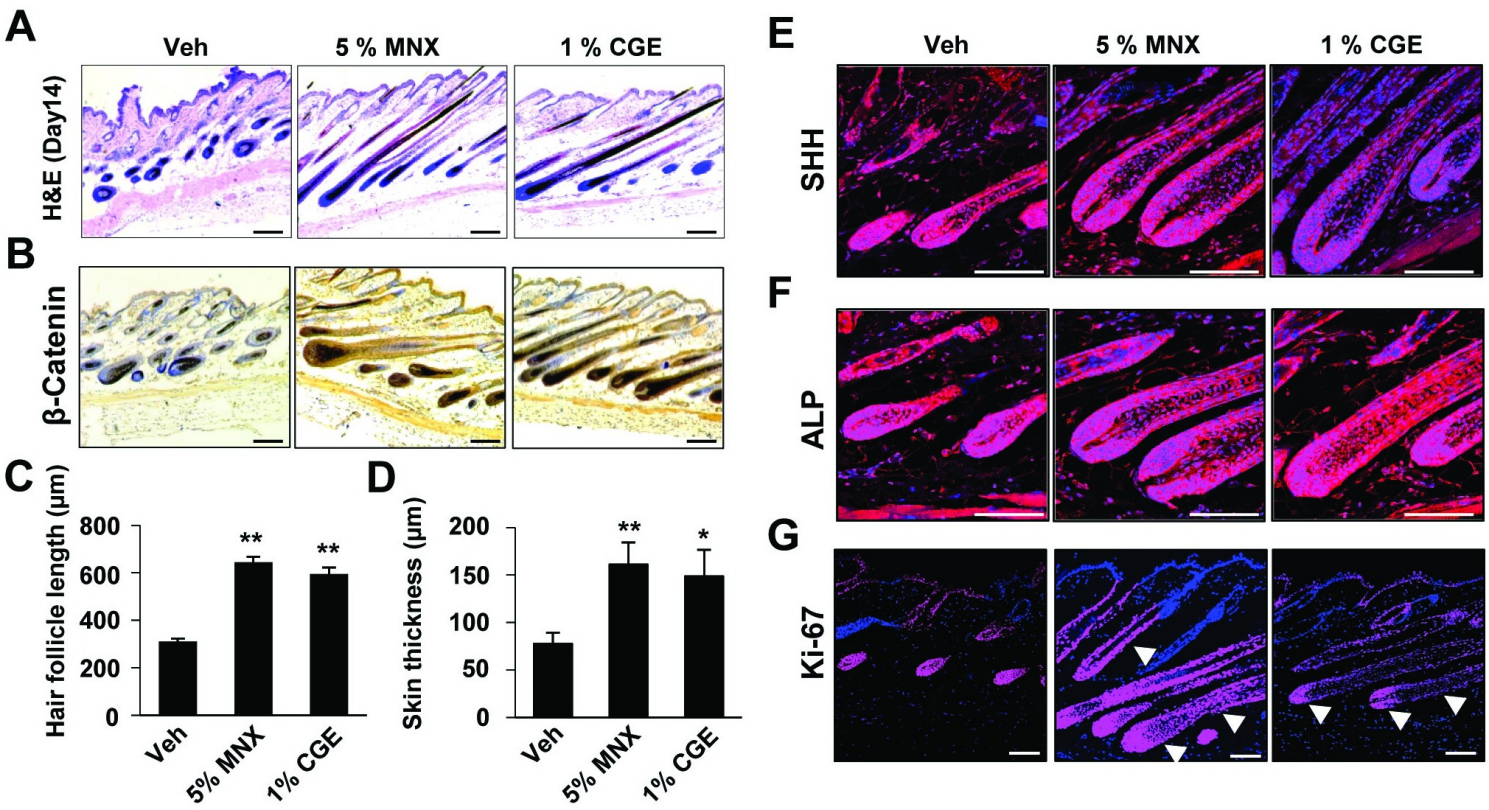
CGE treatment accelerated entry into the anagen phase via up-regulating β-catenin signaling in vivo. (A) Representative images of H&E staining. IHC and IF were conducted for observing (B) β-catenin, (E) SHH, (F) ALP, and (G) Ki-67 expression in mouse skin tissues. Scale bar: 100 μm. Histological quantitative score, (C) hair follicle length and (D) skin thickness were measured for evaluating anagen properties. Data are presented as the mean ± SD. *p ≤ 0.05, **p ≤ 0.01 vs. Veh. Veh, vehicle control; MNX, 5% minoxidil; SD, standard deviation.

To determine whether the effects of CGE promote the induction of anagen hair cycle, we topically administered CGE on depilated mouse dorsal skin; daily topical treatments of 5% MNX were used as a positive control. We observed that CGE causes the diffuse darkening of the dorsal skin, while the Veh group showed weak changes at the 11th day of treatment (Fig 3A). On the 14th day of treatment, hair regrowth was almost completed in the CGE group. Although the dorsal skin in the minoxidil-treated and CGE groups had an incomplete pigmentation and contained hairs in the early stage of the hair cycle, the dorsal skin of the Veh group retained large white and pink skin areas without anagen induction as measured by hair growth score (Fig 3B). Histological data revealed that HFs that were treated with CGE was transformed from the telogen phase to the early- and middle-anagen phases on the 14th day. In the representative longitudinal section (Fig 4A), the CGE-treated group appeared that had an increase in length of HFs and skin thickness compared to those in the Veh group (Fig 4C and 4D). Interestingly, the CGE group was enter the anagen phase earlier with the same tendency as the MNX-treated group. Furthermore, IHC and IF data revealed that the increased expression of the β-catenin protein (a positive regulator of hair growth) (Fig 4B), Sonic hedgehog (SHH, regulators of hair follicular growth and cycling, and act as anagen-inducing signaling molecules) (Fig 4E), and ALP (anagen hair cycle marker) in 1% CGE treatment group compared to Veh group (Fig 4F). Notably, the presence of high expression of Ki-67 was observed in all the matrix and outer-root sheath regions of hair follicle (white arrowhead, Ki-67 expression suggests an active cell cycle and an anagen phase of the hair follicle) in CGE-treated group, but the expression was weaker in Veh group (Fig 4G). These results suggested that CGE treatment accelerated entry into the anagen phase, and the beta-catenin pathway is related to the exerted hair growth in animal models.

## Discussion

Here, we investigated the effects of CGE on hair growth. We observed that CGE enhanced the proliferation of hDPCs and keratinocytes via β-catenin activation, and up-regulation of anagen phase characteristics. In addition, CGE advanced the entry time of hair growth phase by allowing an early onset of anagen in the synchronized telogen mice model. These findings demonstrated that CGE might be enabled to encourage hair follicle growth via β-catenin activation.

The proportion of appearance among modern people’s interests continues to increase, and regardless of age or gender, attention has been focusing on skin, hair, etc. For personal appearance management, hair loss is also a factor influencing an individual’s impression; besides, mental stress from hair loss can be extended to social problems [25]. As already mentioned, minoxidil and finasteride are in the spotlight as treatments for hair loss, but each has limitations due to their adverse effects [26,27]. Recently, several groups have attempted to treat hair loss using stem cells or their secretory factors [28]. However, those sophisticated methods such as stem cell injection or transplantation, cannot be applied in person, so it could be secondary hurdles other than the disease because patients have to put a lot of effort into managing the symptoms. In the context of this, the development of candidates that avoid each limitation (reversible effects) while pursuing existing application methods is a very important issue. The evaluation of the bio-activities of maysin and its derivative flavonoid compounds has been around for a long time. This has been confirmed to have anti-cancer [2], free-radical scavenging activity [3], and anti-inflammatory effects [29], and the therapeutic potential using this is highly appreciated. Therefore, we believe that the biomedical components of maysin and its derivative flavonoid compounds may serve to compensate for conventional treatments.

Hair growth is a dynamic interaction between the dermal and epidermal components, and the dermal papilla cells direct the hair follicle formation of epidermal components [30]. hDPCs are master regulators for the activation of HFSCs, and the active proliferation of hDPCs is necessary for the creation of HF and maintenance of anagen [31,32]. The activation of the HFSCs by the hDPCs allows its proliferation and modification into derived matrix cells of keratinocytes that further differentiate hair shaft and other HF compartments during the anagen phase [33]. To delineate the underlying mechanism of the hair growth effects of CGE, we observed the molecules related to anagen induction associated with HF growth in hDPCs and HaCaT cells. Diverse hair growth-related factors that induced proliferation of cells-constituting HF were established in previous reports (MAPK-ERK1/2, PI3K-AKT signaling, and Ki-67 activation, etc.) [34–36]. Activation of ERK1/2 is reported to enhance cell growth of hDPCs and keratinocytes [37]. AKT is another pathway that controls the signal cascades responsible for cell division and has been shown to guide the anti-apoptosis and proliferation of hDPCs [38]. Here, we have confirmed that CGE not only increased levels of pAKT in hDPCs and HaCaT cells, but also increased the expression of pERK in HaCaT cells. β-catenin is the target molecule for the initiation of HF development [39]. A previous report explained that increased GSK-3β (activation form) promoted β-catenin downregulation in hDPCs isolated from AGA patients [40]. In contrast, β-catenin overexpression facilitates the telogen to anagen transition in mice [41]. Our findings showed that CGE increased CTNNB1 mRNA and β-catenin protein in hDPCs. CGE also promoted β-catenin expression in keratinocytes. Moreover, the IHC results showed that CGE topical treatment increased β-catenin expression in HF tissue compared to the Veh group. Previous studies elaborated on the crosstalk between β-catenin and Akt/ERK1/2 pathways, and their involvement in hair growth promotion [42–44], and β-catenin expression is promoted by pGSK3β-pAKT-pERK signaling in hDPCs [45]. A previous study reported that β-catenin regulates SHH expression, and the deletion of β-catenin inhibited SHH expression and inhibited anagen induction [39]. Consistently, CGE administration increased SHH expression in mice hair follicles in this study. Altogether, these results suggested that CGE promoted the proliferation of cells which constitutes HF tissue, through the enhancement of β-catenin signaling, which mediates the telogen to anagen transition and hair regrowth.

In summary, CGE increased hair anagen properties in hDPCs and keratinocytes and promoted growth phase entry in mouse animal models. Importantly, β-catenin was observed to play a central role in the promotive effects of CGE in hair growth. Based on these current findings, we suggest that the CGE can be used as a therapeutic reagent for hair loss as potentially. We suggest that CGE may be a potential treatment to enhance hair growth and prevent hair loss. However, it may be the most important part to find the crucial factor in hair growth promotion among the CGE components, and this limitation needed to be explored in further study.

## Supporting information

S1 Fig (PPTX)

S2 Fig (PPTX)

S1 Table List of primer sequences used for RT-PCR analysis in this study. (DOCX)

## Abbreviations

AKT: protein kinase B
ALP: alkaline phosphatase
ANOVA: One-way analysis of variance
CGE: centipedegrass extract
CTNNB1: catenin beta 1
GSK3β: glycogen synthase kinase 3β
hDPCs: human hair dermal papilla cells
HF: hair follicle
IF: immunofluorescence
IGF1: insulin-like growth factor 1
IHC: Immunohistochemistry
PCNA: proliferating cell nuclear antigen
SOX2: sex-determining region Y-box 2
VEGFA: vascular endothelial growth factor A
