# Supplementary figures and images for: The effects of centipedegrass extract on hair growth via promotion of anagen inductive activity
Source: PLoS One. 2022 Mar 23;17(3):e0265532. doi: 10.1371/journal.pone.0265532 (PMC8942214; doi:10.1371/journal.pone.0265532)

## Slide 1
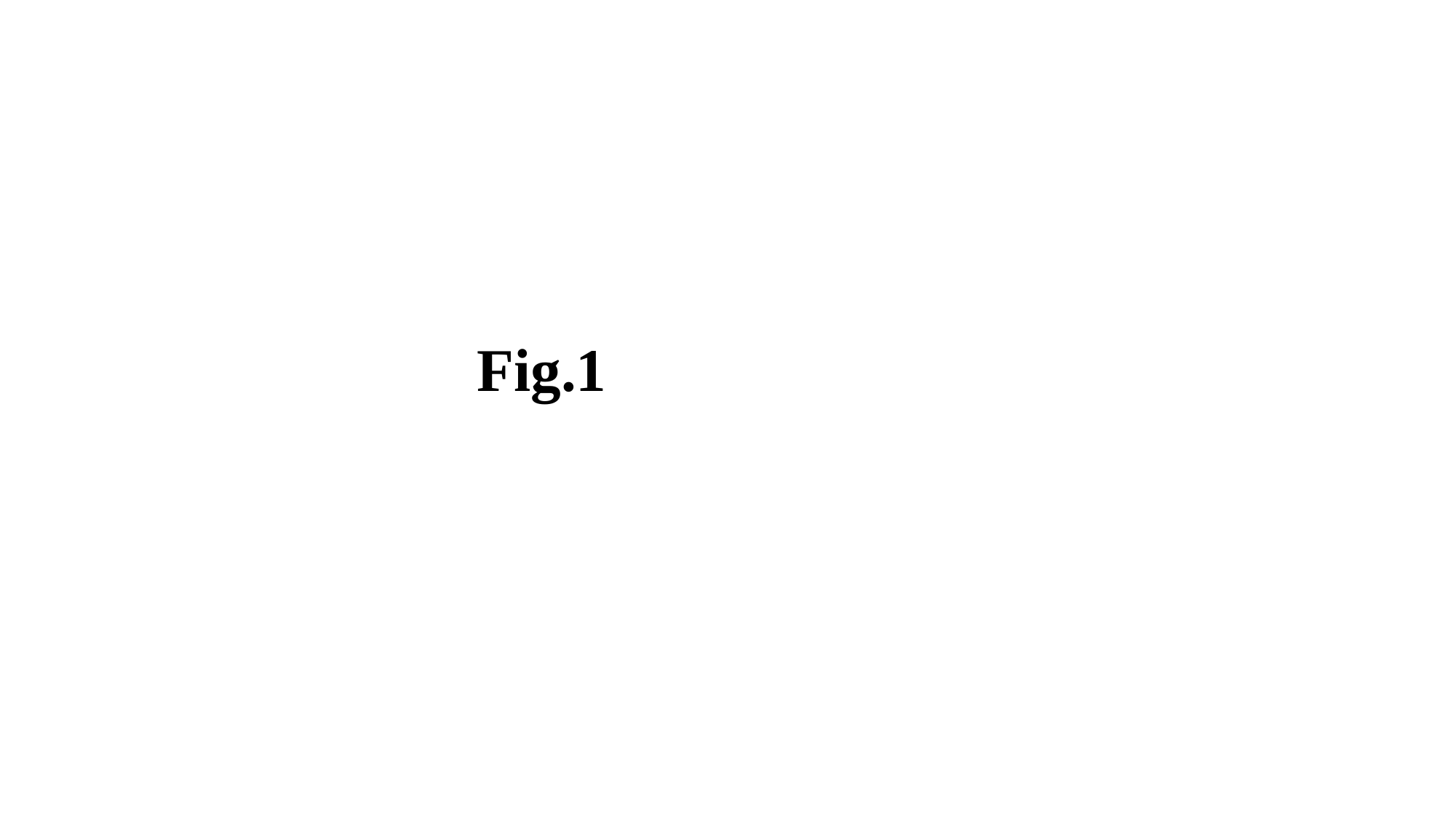

Fig.1

## Slide 2
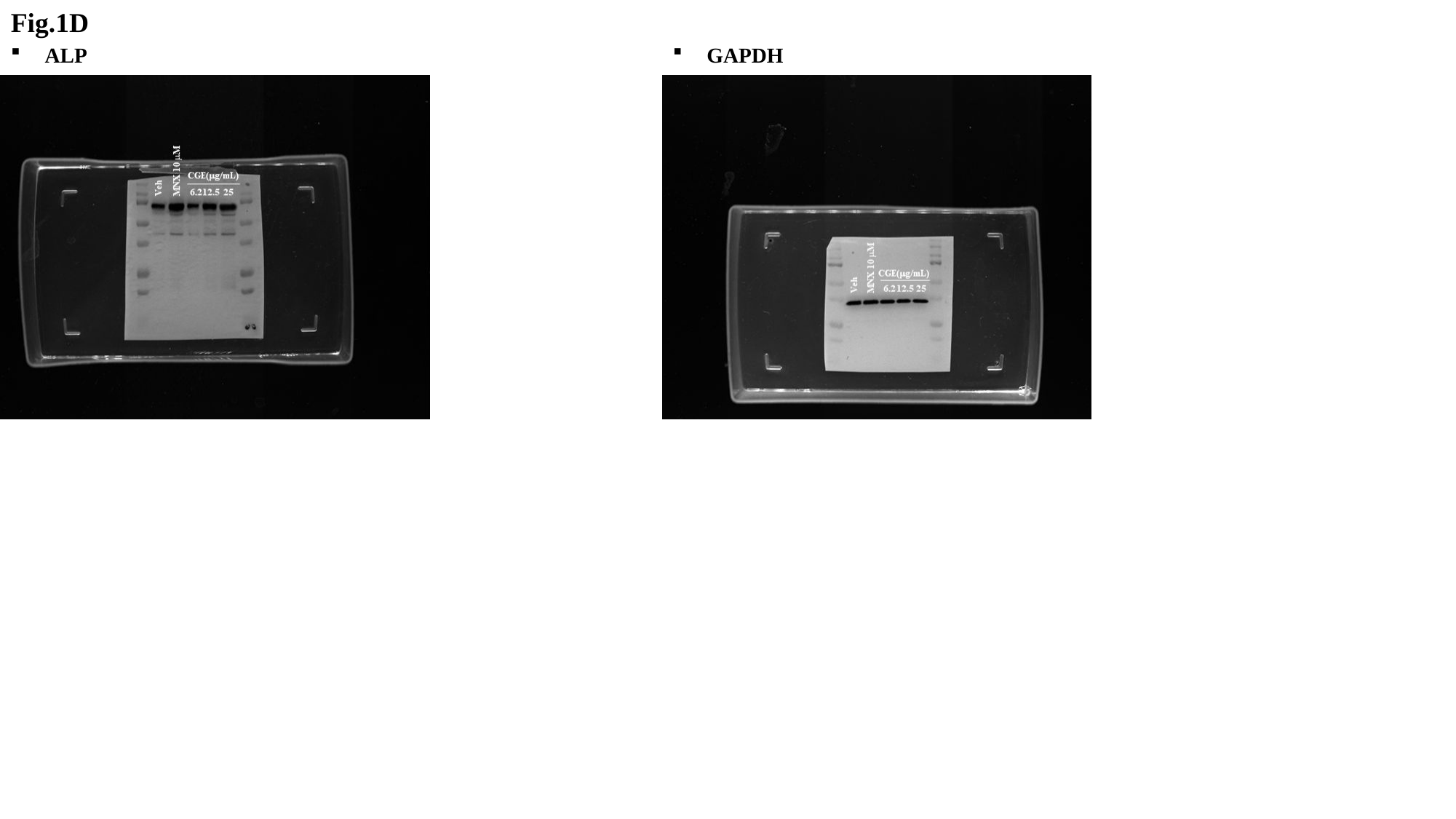

Fig.1D
GAPDH
ALP

## Slide 3
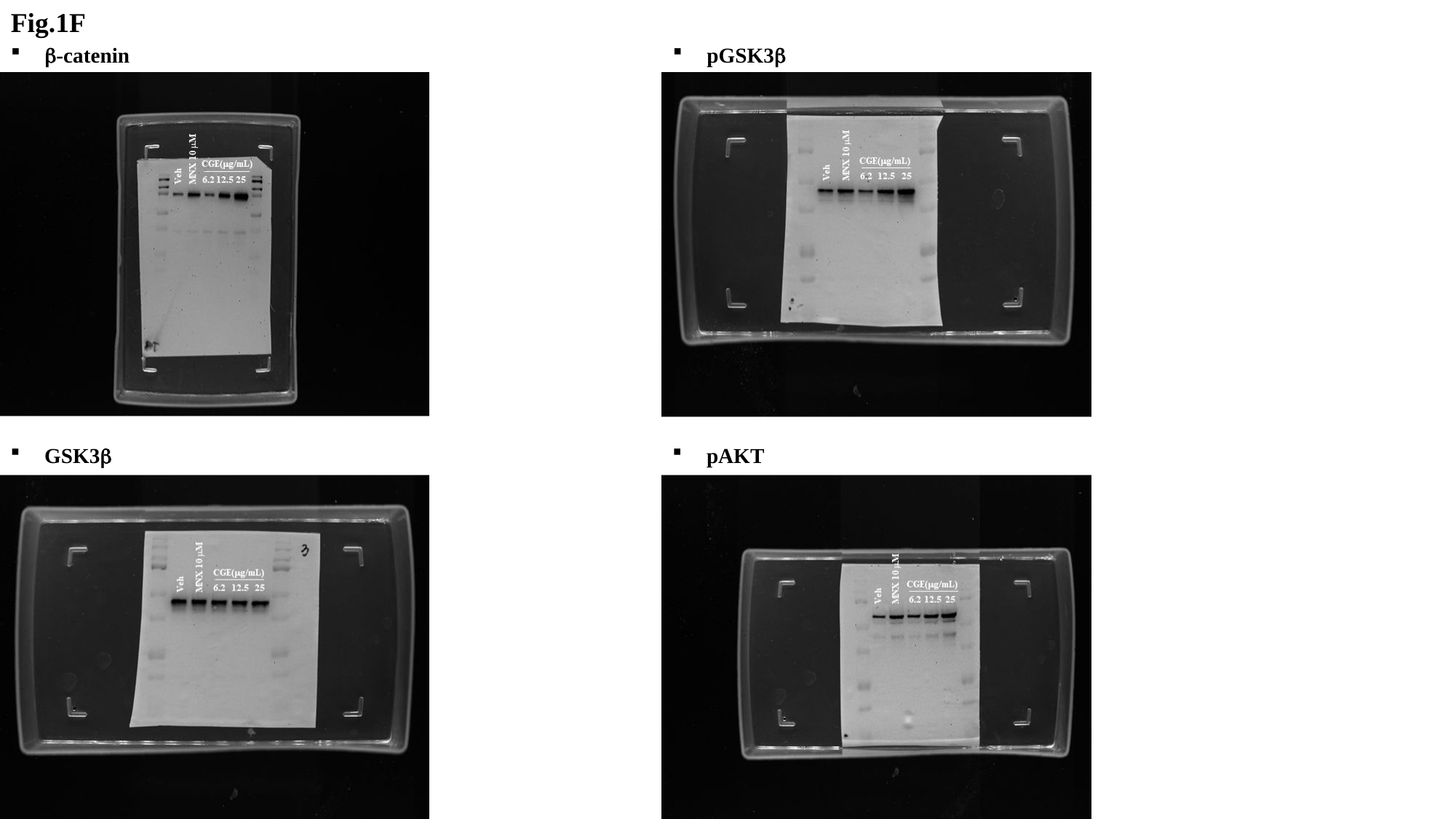

Fig.1F
pGSK3β
β-catenin
pAKT
GSK3β

## Slide 4
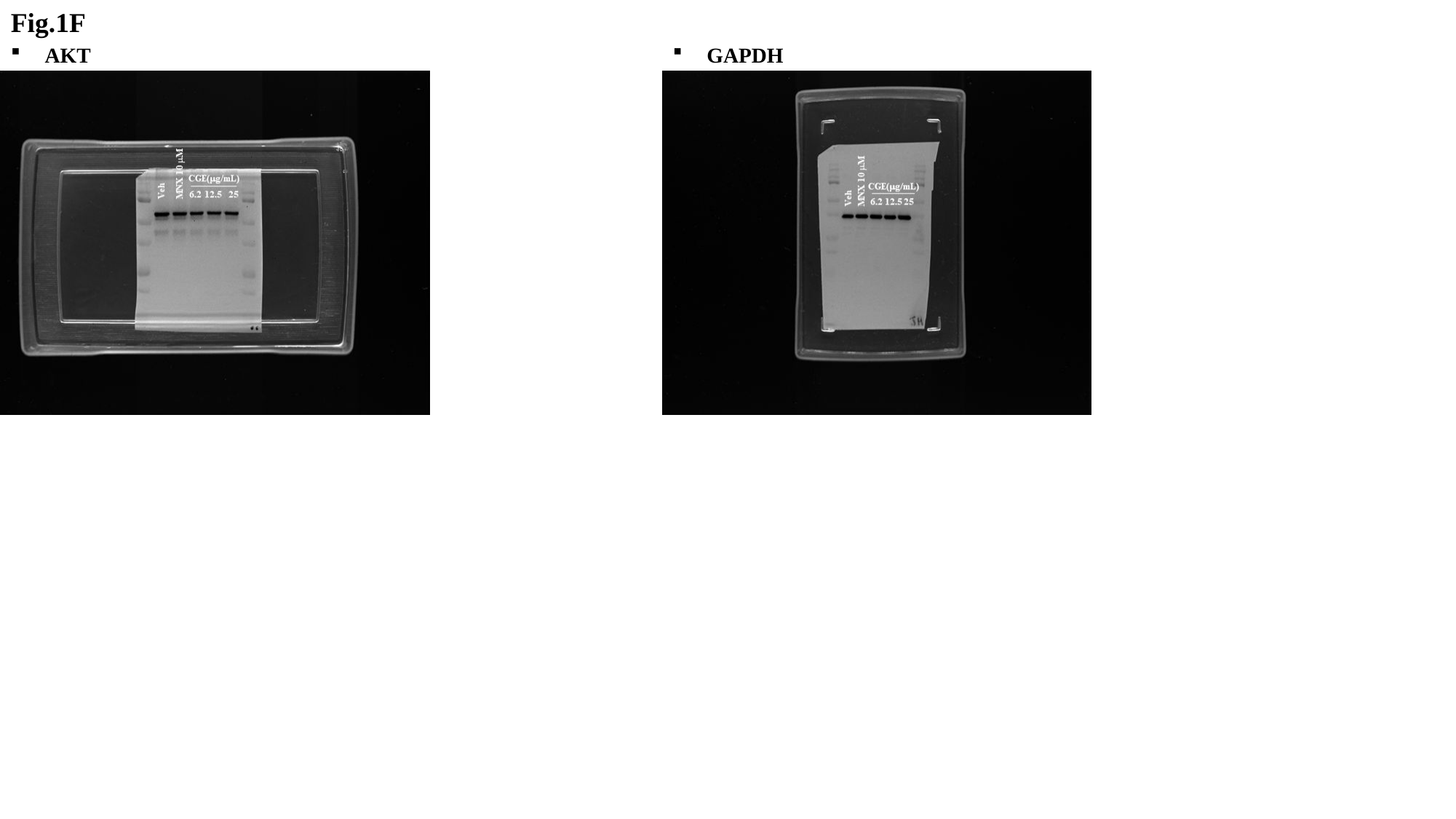

Fig.1F
GAPDH
AKT

Supplement: S1 Fig — (PPTX) [file pone.0265532.s001.pptx]

## Slide 1
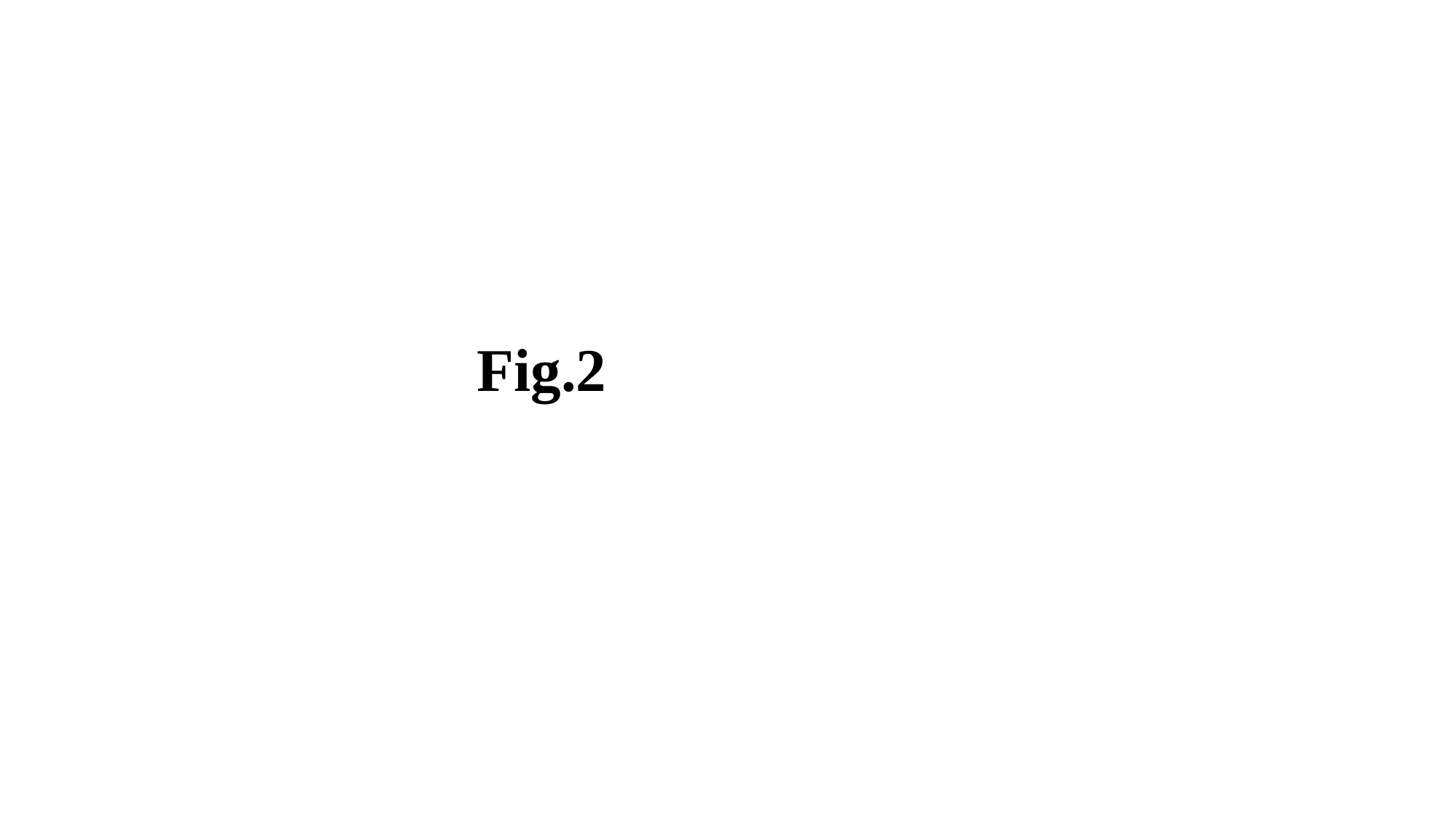

Fig.2

## Slide 2
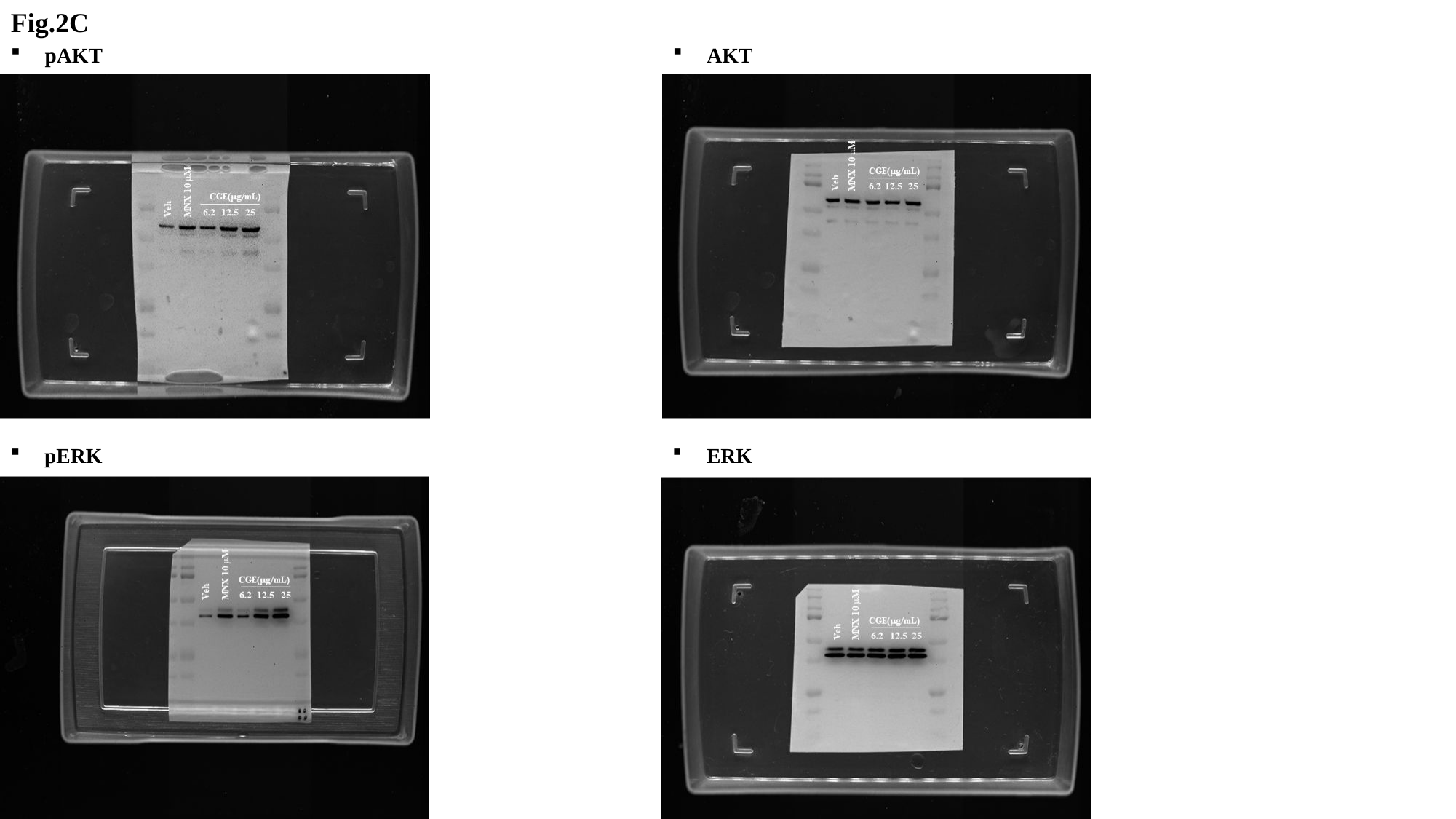

Fig.2C
AKT
pAKT
ERK
pERK

## Slide 3
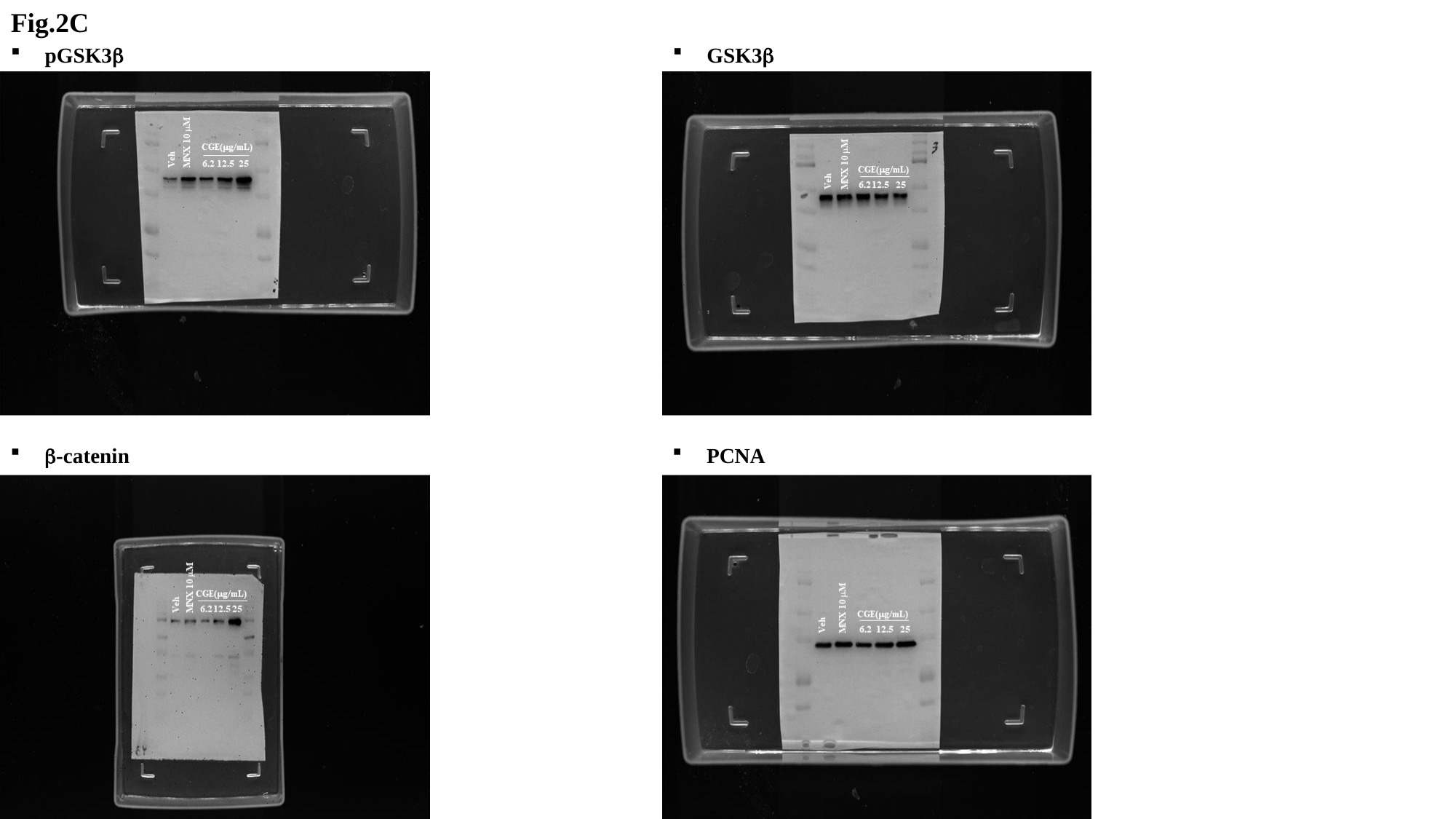

Fig.2C
GSK3β
pGSK3β
PCNA
β-catenin

## Slide 4
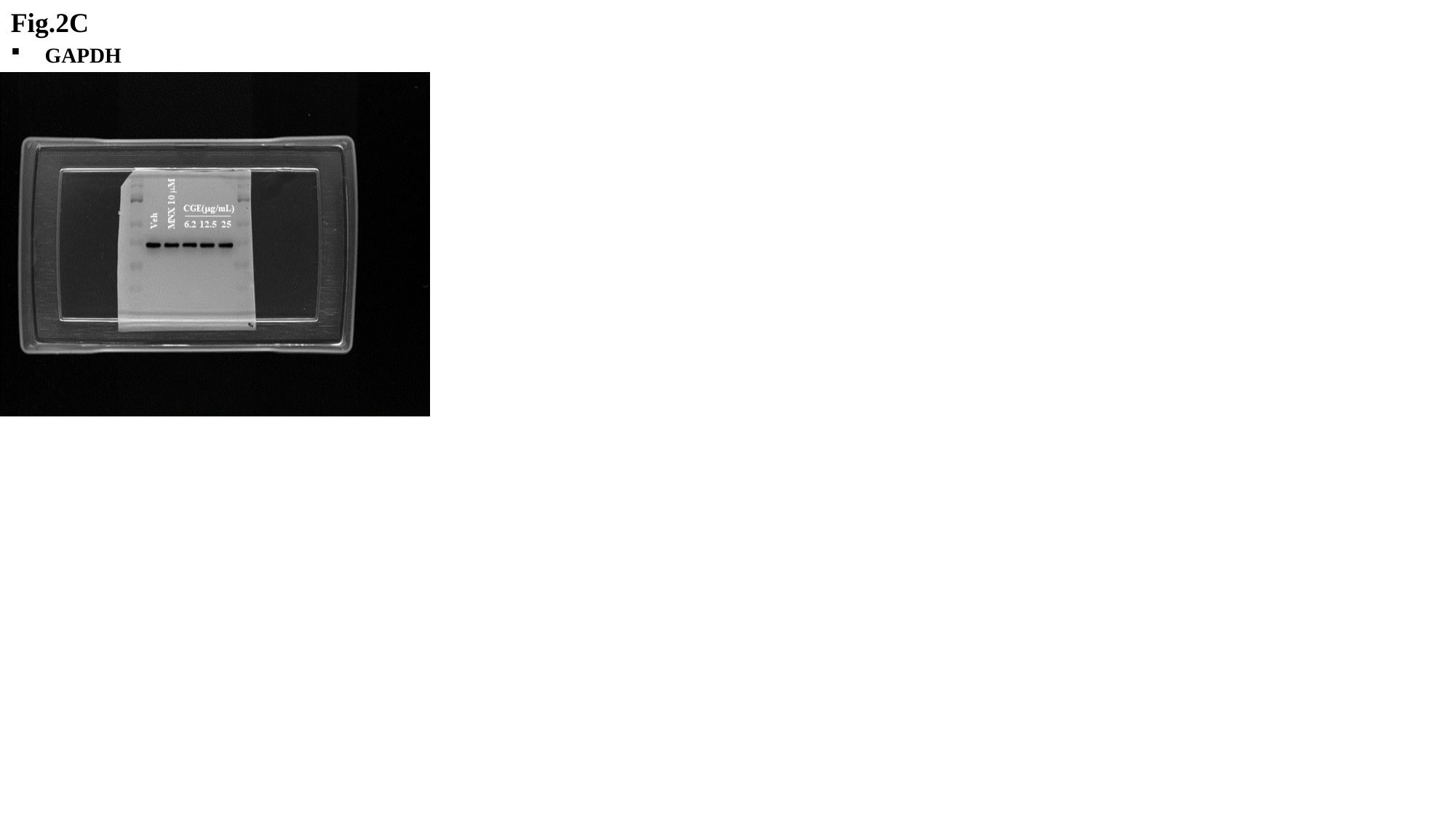

Fig.2C
GAPDH

Supplement: S2 Fig — (PPTX) [file pone.0265532.s002.pptx]
