# Supplementary material for: The effects of centipedegrass extract on hair growth via promotion of anagen inductive activity
Source: PLoS One. 2022 Mar 23;17(3):e0265532. doi: 10.1371/journal.pone.0265532 (PMC8942214; doi:10.1371/journal.pone.0265532)
Supplement: S1 Table — (DOCX) [file pone.0265532.s003.docx]

| Genes |  | Sequences (5′-3′) |
| --- | --- | --- |
| *GAPDH* | Forward | CCA CTC CTC CAC CTT TGA CG |
|  | Reverse | CCA CCA CCC TGT TGC TGT AG |
| *SOX2* | Forward | AGA ACC CCA AGA TGC ACA AC |
|  | Reverse | CGG GGC CGG TAT TTA TAA TC |
| *CTNNB1* | Forward | GCC GGC TAT TGT AGA AGC TG |
|  | Reverse | GAG TCC CAA GGA GAC CTT CC |
| *ALPL* | Forward | CCT CCT CGG AAG ACA CTC TG |
|  | Reverse | AGA CTG CGC CTG GTA GTT GT |
| *IGF1* | Forward | TGG ATG CTC TTC AGT TCG TG |
|  | Reverse | TGG TAG ATG GGG GCT GAT AC |
| *VEGFA* | Forward | TGA GGA GTC CAA CAT CAC CA |
|  | Reverse | TTT CTT GCG CTT TCG TTT TT |
